# Revealing the Dynamic History of Parasitic Plant Plastomes via Structural Characterization, Comparative Analysis, and Phylogenomics

**DOI:** 10.3390/genes15121577

**Published:** 2024-12-08

**Authors:** Lubna, Sajjad Asaf, Rahmatullah Jan, Saleem Asif, Saqib Bilal, Kyung-Min Kim, In-Jung Lee, Ahmed AL-Harrasi

**Affiliations:** 1 Natural and Medical Science Research Center, University of Nizwa, Nizwa 616, Oman; lubnabilal68@gmail.com (L.); saqib@unizwa.edu.om (S.B.); aharrasi@unizwa.edu.om (A.A.-H.); 2 Coastal Agriculture Research Institute, Kyungpook National University, Daegu 41566, Republic of Korea; rehmatbot@yahoo.com; 3 Department of Applied Biosciences, Kyungpook National University, Daegu 41566, Republic of Korea; saleemasif10@gmail.com (S.A.); ijlee@knu.ac.kr (I.-J.L.)

**Keywords:** parasitic plant, genome reduction, gene loss, gene divergence, phylogenetic study

## Abstract

**Background:** The shift to a parasitic lifestyle in plants often leaves distinct marks on their plastid genomes, given the central role plastids play in photosynthesis. Studying these unique adaptations in parasitic plants is essential for understanding the mechanisms and evolutionary patterns driving plastome reduction in angiosperms. By exploring these changes, we can gain deeper insights into how parasitism reshapes the genomic architecture of plants. **Method:** This study analyzed and compared the plastomes of 113 parasitic plants from different families. **Results:** The Orobanchaceae family (hemiparasitic plants) displayed the largest plastome size, while Apodanthaceae exhibited the shortest. Additionally, Orobanchaceae showcased little to no gene loss in their plastomes. However, holoparasitic species typically had reduced plastome sizes. Convolvulaceae exhibited significantly reduced plastome sizes due to high gene loss, and Apodanthaceae retained only a few genes. Gene divergence among different families was also investigated, and *rps15, rps18,* and *rpl33* in Orobanchaceae; *acc*D and *ycf1* in Convolvulaceae; *atp*F and *ccs*A *i*n Loranthaceae; and *rpl32* in Santalaceae showed greater divergence. Additionally, Orobanchaceae had the highest numbers of all repeat types, whereas Loranthaceae and Convolvulaceae exhibited the lowest repeat numbers. Similarly, more simple sequence repeats were reported in Loranthaceae and Santalaceae. Our phylogenetic analysis also uncovered a distinct clade comprising Loranthaceae, with a single Schoepfiaceae species clustering nearby. Contrary to expectations, parasitic and hemiparasitic plants formed mixed groupings instead of segregating into separate clades. **Conclusions:** These findings offer insights into parasitic plants’ evolutionary relationships, revealing shared and divergent genomic features across diverse lineages.

## 1. Introduction

Although green plants are often collectively seen as primary producers, the process of photosynthesis has been lost repeatedly, most often in flowering plants. Approximately 4500 parasitic species have been documented across 20 families of flowering plants [1,2]. True parasitism, which necessitates the development of direct vascular connections between heterotrophic plants and their hosts, has independently evolved at least 12 times throughout the evolutionary tree of angiosperms [3]. The transition from photosynthetic ancestors to parasitic plants is frequently accompanied by significant morphological, physiological, and genomic changes. These adaptations are driven by a decreased or entirely absent reliance on the photosynthetic apparatus [4,5,6,7]. Moreover, the genomic changes associated with the transition to a parasitic lifestyle are expected to be particularly evident in plastid genomes because plastids are crucial sites for photosynthesis within plant cells. As plants evolve away from photosynthesis, significant modifications and reductions in plastomes are likely to occur, making plastomes a key focus for studying the genomic effects of transitioning to a parasite [6,7].

Compared to mitochondrial and nuclear genomes, the plastome is notably gene dense. Roughly 50% of the plastid genome is allocated to gene sequences, while the remaining portion consists of introns, regulatory regions, and intergenic spacers [8,9]. As previously mentioned, variations in plastome gene composition and order are rare in autotrophic plants. This is primarily attributed to the stabilizing selective pressure exerted by the necessity for photosynthesis. In contrast, in heterotrophic plants, the selective pressure related to photosynthesis is either significantly reduced or completely lifted. This relaxation of selective constraints allows the plastome to undergo evolutionary changes more freely. The published parasitic plastomes exhibit a wide range of sizes, from nearly intact genomes of around 160,911 bp in the hemiparasitic *Schwalbea americana* (Orobanchaceae) [10] to the shortest assembled example of approximately 11,348 bp in the holoparasitic *Pilostyles aethiopica* (Apodanthaceae). The plastome of *P. aethiopica* is interesting for containing only five genes, none of which are involved in photosynthetic processes [11]. Holoparasitic plants, which are entirely reliant on their host for nutrients, are expected to have more drastically reduced plastomes compared to those of hemiparasitic plants. This is due to the holoparasites’ significantly diminished (or completely absent) reliance on the photosynthetic genes encoded in plastomes. Regarding hemiparasitic plants, one can anticipate that obligate hemiparasites, which primarily rely on parasitism as their main strategy for nutrient acquisition, would likely exhibit more pronounced reductions in their plastomes compared to facultative hemiparasites. Facultative hemiparasites, on the other hand, utilize parasitism as an additional means of obtaining nutrients alongside photosynthesis, which may result in comparatively fewer reductions in their plastomes. These reductions in plastid coding sequences and the loss of functional genes are generally considered irreversible processes [12]. Furthermore, as plants continue to adapt to their heterotrophic lifestyle, their plastomes are expected to undergo a continuous reduction [13].

As an increasing number of heterotrophic plastomes are documented, collectively offering more statistical power, various models have been proposed to describe the progression of changes in gene content that occur following the loss of photosynthesis [14,15,16]. The proposed models anticipate a sequence of events in the evolution of plastid genes in heterotrophic plants. They predict an initial relaxation of purifying selection, followed by the pseudogenization and eventual loss of genes. These events occur in five distinct consecutive categories of plastid genes, which become progressively more central to plastome function [15,17,18]. Regarding plastid gene loss in heterotrophic plants, the *ndh* gene family responsible for mitigating the effects of photo-oxidative stress is usually among the first to be lost. Subsequently, the next phase typically involves the loss of a substantial portion of the photosynthesis genes like *psa*, *psb*, *rbc*L, and *pet*. As the evolutionary process continues, even the essential “housekeeping” genes, *rps*, *rpl*, *rrn*, and *trn*, which provide the cellular machinery necessary for plastid gene expression, may eventually be lost [12,19]. Compared to other photosynthetic genes, the *atp* gene family is typically retained in plastomes for a longer duration. This is because these genes have a secondary, non-photosynthetic function related to protein transport across the thylakoid membrane [20]. Even in heavily reduced plastomes, one or more of the five plastid genes with essential non-bioenergetic and non-housekeeping functions (*acc*D, *clp*P, *trn*E, *ycf1*, and *ycf2*) are retained [10,11]. Moreover, these genes are hypothesized to be the last to persist during the degradation of a typical heterotrophic plastome [16,21].

In the current study, our original goal was to study parasitism by analyzing the plastome’s content and structure. We aimed to (1) determine structural shifts, gene loss, and/or pseudogenization events in parasitic plants by comparing different parasitic lineages; (2) investigate the extent and progression of the plastome reduction in hemiparasites and holoparasites; (3) find gene divergence among different parasitic plant families; and (4) study the phylogenetic relationship of these parasitic plants.

## 2. Materials and Methods

### 2.1. Genome Sampling

The complete plastomes of 113 parasitic species were downloaded (as of 18 September 2023) from the NCBI database (https://www.ncbi.nlm.nih.gov/genome; accessed on 20 September 2023). Species with incorrect gene annotations were subjected to reannotation with two widely used tools, CPGAVAS2 [22] and GeSeq (https://chlorobox.mpimp-golm.mpg.de/geseq.html; accessed on 20 September 2023). Additionally, tRNAscan-SE [23], a well-established program, was employed to identify tRNA genes within the plastomes. To ensure the accuracy of the annotations, a comparative analysis was conducted by comparing the plastomes with reference genomes using Geneious Pro v.10.2.3 [24] and tRNAs can-SE (v.1.21) [23]. The comprehensive details of the 113 chloroplast genomes, including species name, family name, order name, genome size, and accession number, are conveniently listed in Table S1. Graphical representations of the data were generated using the Seaborn library Python package [25].

### 2.2. Analysis of Repetitive Sequences and Simple Sequence Repeats (SSRs)

The identification of repetitive sequences, including direct, reverse, and palindromic repeats within the plastomes, was conducted using REPuter [26]. REPuter was configured with the following settings for repeat identification: a minimum repeat size of 30 bp, a sequence identity threshold of at least 90%, and a Hamming distance of 1. Tandem Repeats Finder version 4.07b was employed to detect tandem repeats, and the analysis was conducted with the default settings online tool Tandem Repeats Finder v.4.09 [27]. To identify SSRs, MISA software (version 2.1) [28] was employed, and the search parameters were configured as follows: a minimum of ≥3 repeat units for pentanucleotide and hexanucleotide repeats, ≥4 repeat units for trinucleotide and tetranucleotide repeats, ≥8 repeat units for dinucleotide repeats, and ≥10 repeat units for mononucleotide repeats.

### 2.3. Genome Divergence

We assessed the variation in shared protein-coding genes (PCG) within the same family. A comparative analysis was executed through multiple sequence alignment, wherein the examination and analysis of gene order were undertaken to enhance the precision of deficient and ambiguous gene annotations. Gene alignment was conducted using MAFFT version 7.222 [29], employing default parameters. Pairwise sequence divergence was calculated utilizing Kimura’s two-parameter (K2P) model [29].

### 2.4. Phylogenetic Study

To resolve the phylogenetic position of parasitic plants, complete genomes from 113 plastomes representing 11 different families were used for the analysis. The nucleotide sequences of these plastomes were aligned using MAFFT, employing the default settings as outlined by Katoh and Standley 2013 [30]. The aligned sequences were then trimmed using trimAl v1.4 [31] with a threshold of 0.7 to prune the aligned data. After trimming, the best-fitting model of nucleotide evolution, GTR+G, was determined by jModelTest 2 [32]. A maximum likelihood (ML) tree was then generated using PAUP* 4.0 [33]. The ML tree was created by running 1000 bootstraps, which provided support values for different nodes. Additionally, Figtree [34] was utilized to visually represent the relationships among the parasitic species based on their plastome sequences.

## 3. Results

### 3.1. Plastome Organization and Composition of Parasitic Plants

**Figure 1:**
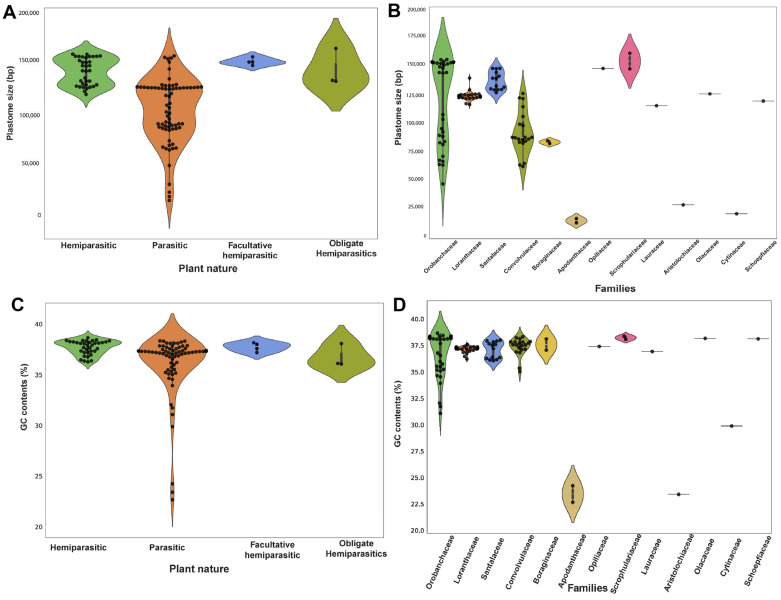
(**A**) Plastome size variation in different parasitic plants based on their nature of parasitism, (**B**) plastome size variation in different parasitic plants based on their families, (**C**) guanine–cytosine (GC) contents across parasitic plants based on their nature of parasitism, (**D**) GC contents across parasitic plants based on their families.

All parasitic plants studied exhibited the typical quadripartite structure consisting of a pair of IRs separated by the large single-copy (LSC) and small single-copy (SSC) regions. The plastome features of 113 parasitic species studied in the current work are presented in Table S1. In this study, the length of the plastome in the parasitic plants varied significantly across the studied species, ranging from 160,910 bp in *Schwalbea Americana* (Orobanchaceae) to only 11,348 bp in *Pilostyles aethiopica* (Apodanthaceae). While analyzing the plastomes of different parasitic families, *S. Americana*, within the family Orobanchaceae, had the largest plastome size, followed by *Brandisia swinglei* (with a plastome length of 155,344 bp), then *Pedicularis cheilanthifolia* (at 155,159 bp), and *Pedicularis longiflora* (at 153,547 bp). Within the Convolvulaceae family, the plastome sizes ranged from 60,959 bp in *Cuscuta erosa* to 125,373 bp in *Cuscuta exaltata*. Among members of the Santalaceae family, *Pyrularia edulis*, *Osyris wightiana*, and *Osyris alba* possessed the longest plastome sizes, measuring 147,530 bp, 147,544 bp, and 147,253 bp, respectively (Figure 1). In contrast, *Viscum crassulae* had the shortest plastome size at about 126,064 bp. Plastome sizes varied significantly in different families. In Loranthaceae, the average plastome size was approximately 123,035 bp, while Opiliaceae exhibited an average plastome size of around 147,457 bp. Similarly, in Boraginaceae, the plastome size averaged approximately 82,436 bp, while in Apodanthaceae, it was notably smaller, with an average size of around 13,257 bp (Figure 1). Other families including Aristolochiaceae, Olacaceae, Schoepfiaceae, Cytinaceae, and Lauraceae had plastome sizes of 27,233 bp (*Hydnora visseri*), 12,505 bp (*Malania oleifera*), 11,8743 bp (*Schoepfia jasminodora*), 19,400 bp (*Cytinus hypocistis*), and 114,622 bp (*Cassytha filiformis)*, respectively. Analyzing the guanine–cytosine (GC) content in parasitic plants revealed a variation from 22% (*Pilostyles hamiltonii*) to 38.6% (*Pedicularis hallaisanensis*). In the Orobanchaceae family, a significant variation in the GC content was observed across all members, ranging from 31% to 38.6%. Moreover, in Convolvulaceae, the variation in the GC content was considerably low (35 to 38%), whereas, in the Santalaceae and Loranthaceae families, the GC content remained relatively constant, with values ranging from 36% to 37%, as displayed in Figure 1. However, the GC content was not evenly distributed across the plastome; the highest GC content was typically found in rRNA regions compared to other parts of the plastome. In our study, the highest GC% was observed in the rRNA of Santalaceae family members like *Phacellaria glomerata* and *Osyris* (55.6%), and the lowest was (29%) in *P. hamiltonii* (Apodanthaceae), as presented in Table S1.

**Figure 2:**
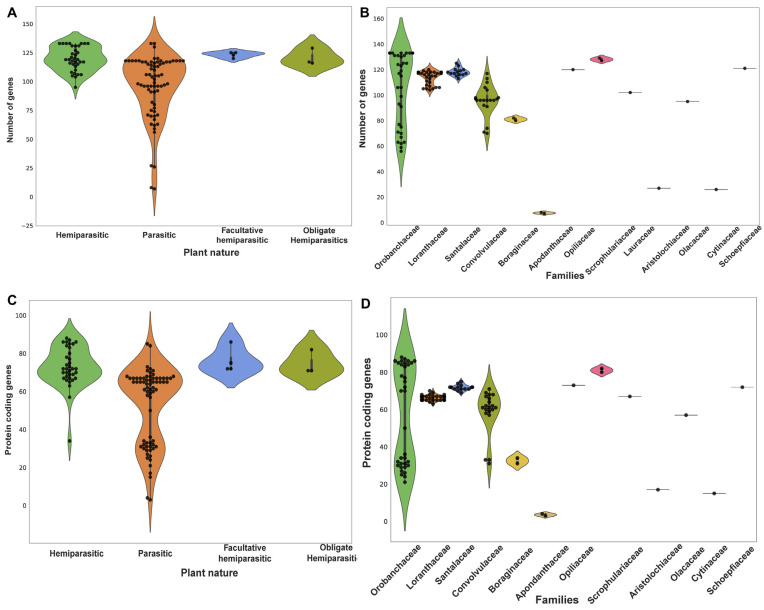
(**A**) Number of genes across parasitic plants based on their nature of parasitism. (**B**) Number of genes across parasitic plants based on their families. (**C**) Number of protein-coding genes (PCG) across parasitic plants. (**D**) Number of PCG in different parasitic plant families.

**Figure 3:**
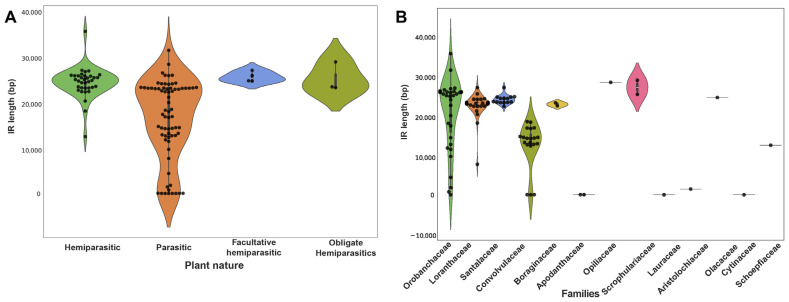
(**A**) The inverted repeat (IR) length across parasitic plants based on their nature of parasitism. (**B**) The IR length in different parasitic plant families.

Overall, the Orobanchaceae family exhibited the longest plastome, while the Apodanthaceae family possessed the shortest plastome. Similarly, the plastomes varied in terms of the number of PCG they contained, ranging from 3 (*P. aethiopica*) to 88 (*P. resupinata*) (Figure 2). Additionally, there was a significant variation within members of the Orobanchaceae family. For example, in *C. Americana*, 21 PCG were identified, whereas in *P. resupinata*, a substantially higher number (88) of PCG were present (Figure 2). The highest PCG number was reported in Orobanchaceae, and the lowest PCG number was studied in Apodanthaceae, with tRNA ranging from 2 to 8 and rRNA ranging from 0 to 39. Additionally, PCG lengths revealed huge variations across different parasitic plant families. Specifically, in *P. resupinata* (Orobanchaceae), the PCG lengths were the longest among the species examined (80,277 bp), contrary to those of *P. aethiopica*, where the PCG lengths were the shortest (1872 bp) (Figure 2). Upon analyzing the IR length, it was directly proportional to the PCG. The greater the number of PCG, the longer the IR length (e.g., the *S. americana* IR length was 28,629 bp, and the number of PCG was 82 and vice versa) (Figure 3).

### 3.2. Gene Deletion and Duplication

**Figure 4:**
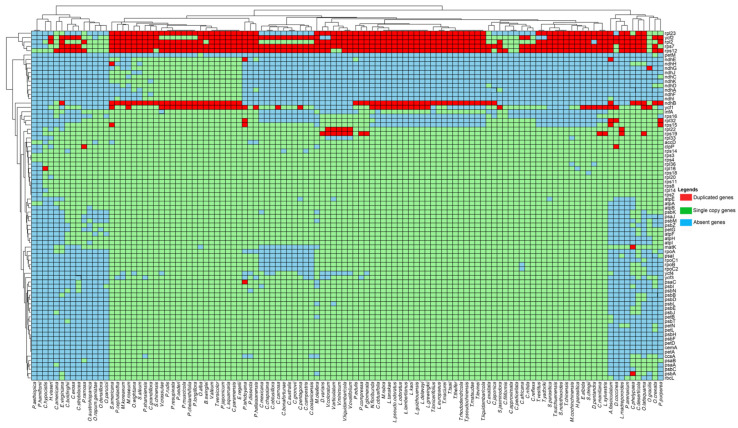
Summary of gene loss across parasitic plant plastomes. The blue color shows missing genes, the green color shows single genes, and the red color shows the duplicated genes in these plastomes.

These results confirm the expectation of substantial variation in terms of genome size and composition within parasitic plant families. Compared to the plastomes of autotrophic plants, those of parasitic plants commonly exhibit gene losses. In this study, the entire *ndh* gene family, including *ndh*A, B, C, D, E, F, G, H, I, J, and K, has been nearly lost in most studied parasitic plant families. However, within the family Orobanchaceae, as well as in other related families like Santalaceae, some specific species including *Brandisia swinglei*, *Castilleja paramensis*, *Euphrasia regelii*, *Lathraea squamaria*, *Pedicularis cheilanthifolia*, *Pedicularis longiflora*, *Pedicularis muscicola*, *Pedicularis oederi*, *Pedicularis resupinata*, *Pedicularis rudis*, *Pedicularis shansiensis*, *Phtheirospermum japonicum*, *Siphonostegia chinensis*, and *Triphysaria versicolor* in Orobanchaceae and *O. alba*, *O. wightiana*, and *Viscum album* in Santalaceae retained these genes (Figure 4). Additionally, the ribosomal protein-coding *rps7* gene was lost from *C. hypocistis* (Cytinaceae) and the *rpl2* gene, notably in *Dendrophthoe pentandra*, a member of Convolvulaceae, was retained as a single copy. The *ycf2* gene was lost in *Viscum coloratum* and *D. varians* of Santalaceae; *T. vestitus* and *T. yadoriki* of Loranthaceae; *Orobanche rapum-genistae*of Orobanchaceae; and in the Apodanthaceae family. In addition to the previously mentioned gene losses, the *rpl23* gene was also missing in *S. jasminodora* (Schoepfiaceae), along with other families like Apodanthaceae, Convolvulaceae, and certain members of Orobanchaceae, including *Diphelypaea coccinea*, *Orobanche austrohispanica*, *Orobanche densiflora*, *Orobanche pancicii*, and *O. rapum-genistae*. The *ycf15* gene, which has an unknown function, was only present in some species of Orobanchaceae and Loranthaceae, but it was lost in the remaining parasitic species. Similarly, the *ycf1* gene has been lost in all species, whilst only a single copy of the gene was present in Convolvulaceae. Like other genes, the initiation factor gene (*infA*) has been lost in some species of Santalaceae (*Viscum* species), Loranthaceae (*Taxillus* species), and Convolvulaceae. The *rps15*, *rps16*, *rpl32*, *ycf3*, and *ycf4* genes were lost in *P. purpurea*, *Epifagus virginiana*, Orobanchaceae, and Loranthaceae species. Additionally, other genes like *rpl22* were lost in *E. virginiana* and *H. visseri*, and the maturase-encoding *matK* gene was only found in *O. alba* and Convolvulaceae species. Along with *psaI*, the *rpo*A, B, *rpo*C1, and *rpo*C2 genes were missing in *Phelipanche purpurea*, *E. virginiana*, *Cistanche deserticola*, *Lennoa madreporoides*, *C. phelypaea*, *C. americana*, *Orobanche crenata*, *Orobanche latisquama*, *D. coccinea*, *Aphyllon fasciculatum*, Convolvulaceae, and some Orobanchaceae species. Likewise, the *acc*D gene was missing in *Pedicularis hallaisanensis*, *Pedicularis dissecta*, *E. regelii*, *O. austrohispanica*, and *C. hypocistis*, while the *pet*M gene was lost in all studied species except *O. crenata* (Orobanchaceae) and *O. alba* (Santalaceae). Additionally, many of the genes related to cytochrome b6/f (*pet*), photosystem I and II (*psa* and *psb*), and ATP synthase (*atp*) genes essential for the proper functioning of the photosynthetic machinery, including *atp*A, *atp*B, *atp*E, *atp*H, *atp*I, *atp*F, *psa*A, *psa*B, *psa*C, *psa*J, *psb*A, *psb*B, *psbc*C *psb*D, *psb*E, *psb*F, *psb*H, *psb*I, *psb*K, *psb*L, *psb*M*. Psb*N, *psb*T, *psb*Z, *pet*A, *pet*B, *pet*D, *pet*G, *pet*L, *pet*N, *cem*A, *ccs*A, and *rbc*L, have been lost from the plastomes of *A. fasciculatum*, *Diphelypaea coccinea*, *L. madreporoides*, *E. virginiana*, *O. crenata*, *C. phelypaea*, *C. deserticola*, *P. arenarium*, *C. Americana*, *O. latisquama*, *H. visseri*, *C. hypocistis*, *C. erosa*, and *Orobanche* species as a result of these species becoming parasites. In all studied families, members of Apodanthaceae, *P. hamiltonii*, and *P. aethiopica* lost all the mentioned genes except *rps12*, *acc*D, *rps3*, *rps4*, and *atp*A. In this study, alongside gene deletion, gene duplication also occurred, and the *ndh*B gene was duplicated in all species except a few (other than those that lost the *ndh*B gene), while *ndh*E was duplicated in *A. fasciculatum* and *P. ishidoyana* (Figure 4). Similarly, the *rps7*, *rps12*, *rpl2*, *rpl23*, and *ycf2* genes in most of these species were duplicated, with some exclusions. Additionally, *ycf1* and *ycf15* were duplicated in members of Orobanchaceae and Loranthaceae, *rps15* and *rpl32* were duplicated only in *P. purpurea*, and *rpl22* was duplicated in some *Viscum* species. Other genes like *rps19* were duplicated in *C. deserticola*, *D. coccinea*, *Viscum minimum*, *Lepionurus sylvestris*, *Pyrularia edulis*, *Phacellaria compressa*, and four *Viscum* species. Besides this, the *matK* gene was only duplicated in *C. phelypaea*, while the *clp*P, *psa*C, and *psb*A genes were duplicated in *A. fasciculatum*, *V. ovalifolium*, and *C. phelypaea*, respectively, as illustrated in Figure 4.

### 3.3. Comparative Study and Gene Divergence Within Parasitic Plant Families

**Figure 5:**
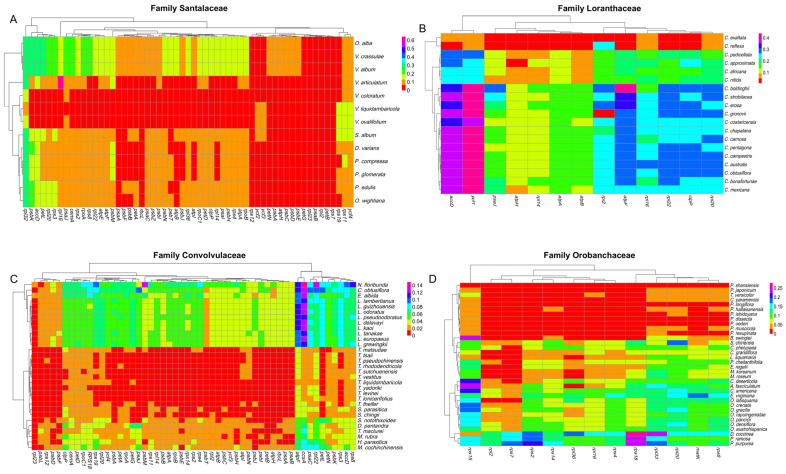
(**A**) Heatmap illustrating pairwise sequence distances of genes within the family Santalaceae plastomes, using *V. minimum* as the reference plastome; (**B**) heatmap illustrating pairwise sequence distances of genes within the family Loranthaceae, using *T. chinensis* as the reference plastome; (**C**) heatmap illustrating pairwise sequence distances of genes within the family Convolvulaceae, using *C. japonica* as the reference plastome; and (**D**) heatmap illustrating pairwise sequence distances of genes within the family Orobanchaceae, using *P. rudis* as the reference plastome.

We calculated the average pairwise sequence divergence for PCG in different parasitic plant families. Analysis of PCG divergence in selected plastomes revealed a distinct pattern, as shown in Figure 5. Within the family Santalaceae, we observed the highest pairwise sequence divergence (0.644) in the *rpl16* gene in the *V. articulatum* species. A significant divergence was also noted in the *rpl32* gene (0.31) across all species within the studied family. Moreover, the *acc*D*, cem*A*, pet*L, *psb*K, *rpl20*, *rps3*, and *rps8* genes in *V. minimum*, *V. crassulae*, and *V. album* exhibited divergence compared to other species (Figure 5A). Similarly, in the Loranthaceae family, the *atp*F (0.1) and *ccs*A (0.13) genes exhibited the highest levels of divergence in the *Loranthus* species along with *Nuytsia floribunda*. The latter species also exhibited divergence in the *acc*D, *psb*M, *rpl22*, and *rps8* genes. In this family, the *E. albida* species exhibited notable divergence in the *ccsA* gene (0.13) showcased in Figure 5B. A significant divergence was observed in the *acc*D (0.38) and *ycf1* (0.43) genes within the family Convolvulaceae. Additionally, the *atp*F gene (0.41) also exhibited a huge genetic divergence within this family (Figure 5C). In Orobanchaceae, the divergence was also high. Specifically, the *rps15*, *rps18*, and *rpl33* genes displayed high divergence while the other genes like *matK*, *rpl2*, *rpl16*, *rpl18*, *rps2*, *rps4*, and *rps8* revealed competitively low divergence, as shown in Figure 5D.

### 3.4. Analyses of Long Repeat Sequences and SSRs

**Figure 6:**
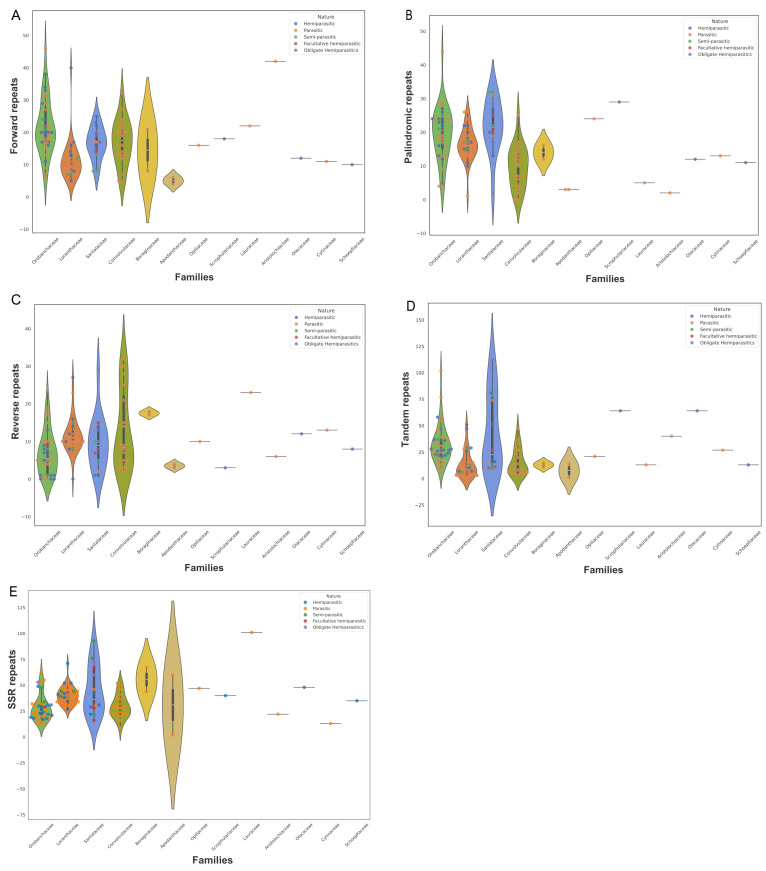
Repetitive sequences in parasitic plants. (**A**) The number of forward repeats across parasitic plant plastomes. (**B**) The number of palindromic repeats across parasitic plant plastomes. (**C**) The number of reverse repeats across parasitic plant plastomes. (**D**) The number of tandem repeats across parasitic plant plastomes. (**E**) The number of simple repeat sequences across parasitic plant plastomes.

The long repeats encompassing forward (F), palindrome (P), and reverse (R) repeats were examined in this study, and the highest total number of repeats was reported in Orobanchaceae, specifically *O. crenata*, with 54 repeats. Contrarily, in Apodanthaceae, the lowest number of repeats was identified with *P. hamiltonii* consisting of only 10 repeats. Most of these repeats were predominantly 18–30 bp in length, as illustrated in Figure 6A. In all studied parasitic plants, the predominant repeat types were palindromic and forward. *P. ramose* exhibited the highest number of forward repeats (46), while *O. rapum-genistae* displayed the highest palindromic repeats (44) in Orobanchaceae (Figure 6). Conversely, *C. approximate* and *C. chapalana* lacked palindromic repeats, while *Scurrula notothixoides*, *Cistanche phelypaea*, *O. rapum-genistae*, *P. cheilanthifolia*, *B. swinglei*, and *P. muscicola* did not possess reverse repeats. In the case of tandem repeats, most of the repeats were 11–20 bp long, and their numbers displayed a wide range of variation from 2 (*Loranthus* species) to 112 (*V. liquidambaricola*). In all families, Santalaceae stood out with the greatest number of tandem repeats, while Loranthaceae displayed the lowest number. Additionally, the SSR analysis in parasitic plants revealed that mononucleotides were the most abundant nucleotide type across all species, followed by dinucleotides. However, tri-, penta-, and hexanucleotides were present in a few species, whereas tetranucleotides were entirely absent in all species. Lauraceae (*C. filiformis)* contained the highest number of SSR (101) repeats (Figure 6E). On the other hand, in Apodanthaceae, represented by *P. hamiltonii*, the number of SSRs diminished to two. Additionally, the most predominant mononucleotide repeats were A/T, and the most common dinucleotide repeat was TA (Figure 6).

### 3.5. Phylogenetic Analyses

**Figure 7:**
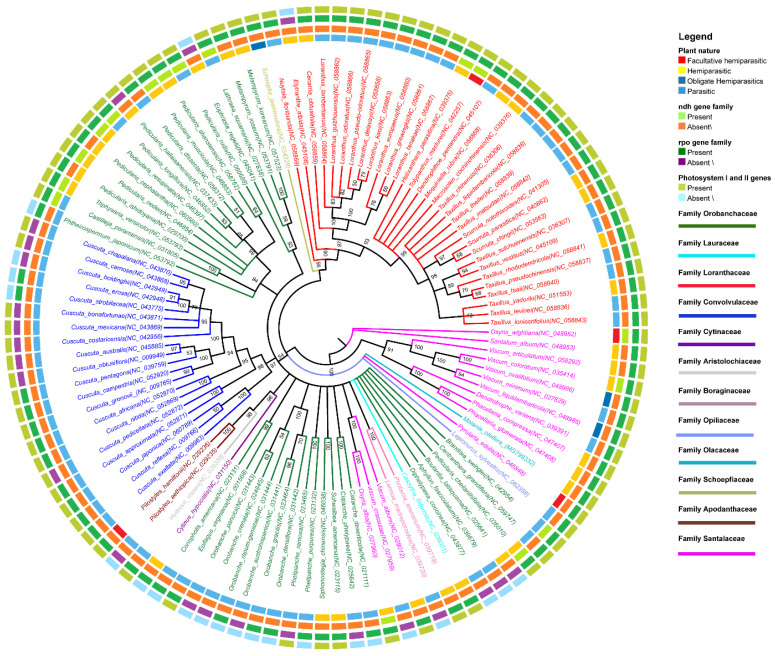
Phylogenetic trees were constructed based on a complete plastome dataset among 113 parasitic plants from 12 different families using the maximum likelihood (ML) method. The numbers above the branches are bootstrap values of ML.

Analyzing the phylogenetic relationships among parasitic plastomes across different families presents significant challenges due to the considerable divergence among these families and the propensity for random gene loss within species of the same genus. Selecting appropriate genes or genomic regions for phylogenetic analysis adds another layer of complexity to the task. Initially, our attempts using a single common gene across all plastomes yielded unsatisfactory results, prompting us to employ the entire plastome for analysis. Notably, members of the Loranthaceae family formed a distinct clade, with one species from the Schoepfiaceae family clustering alongside (Figure 7). Surprisingly, rather than segregating by their parasitic or hemiparasitic nature, the plant species assembled into mixed clades. For instance, all holoparasitic plants from the Convolvulaceae family clustered tightly together in a separate clade with robust bootstrap support. The absence of the entire *ndh* gene family was notable across many parasitic species, contrasting with the sporadic presence of genes from the *rpo* gene family. Additionally, certain genes related to photosystems I and II were identified in select species (*C. boldinghii*, *C. erosa*, and *C. strobilacea*), clustering within the same clade. Unexpected associations between families were also found. Notably, some species from the Santalaceae family clustered alongside members of the Boraginaceae family. Within the largest family, Orobanchaceae, 35 species were divided into two distinct clades, predominantly comprising parasitic species devoid of the *ndh* gene family (Figure 7). Intriguingly, the Apodanthaceae family, characterized by the smallest plastomes, clustered together with *H. visseri* and *C. hypocistis* from the Aristolochiaceae and Cytinaceae families, respectively. Overall, our findings illuminated the intricate evolutionary relationships among parasitic plants, highlighting both shared and divergent genomic features across diverse lineages.

## 4. Discussion

Parasitic plants often possess simplified plant bodies, making it challenging to distinguish them morphologically [35]. The shift from an autotrophic to a heterotrophic lifestyle has had a profound effect on the plastome of parasitic plants. Research on parasitic plant plastomes has revealed a broad spectrum of genomic degradation, primarily characterized by the presence or absence of photosynthetic activity and the extent of their dependence on the host. In the current research, a comparative study of plastomes of different parasitic plants from various families was conducted, and it was observed that plastomes of hemiparasites and holoparasites have undergone structural changes and size reduction. However, it is noteworthy that certain hemiparasitic species within the Orobanchaceae family, such as *S. americana*, have exhibited an exception by possessing the largest plastome of about 160,910 bp. Currently, in all examined genomes, *P. aethiopica* stands out with the most dramatically reduced plastome of about 11,348 bp. Surprisingly, only five genes have been retained in its plastome, while the rest of the genes have been lost. The observation of a higher number of missing genes and a smaller plastome size (Table S1) strongly suggests that gene loss plays a significant role in the reduction of plastome size in parasitic plants.

The genetic changes in the plastomes of hemiparasitic plants are generally less extensive compared to those of holoparasitic plants. However, research on hemiparasitic plants holds great importance in gaining insights into the evolutionary process of transitioning from autotrophs to parasites [36]. The decrease in genome size and gene number in plastomes is observed to occur gradually in correlation with an increasing number of pseudogenization events and gene losses, which can vary in scale among different species. Pseudogenes and gene losses were not revealed in autotrophic plants; however, their presence is observed occasionally in many hemiparasites and, more frequently, in holoparasites [37]. Hemiparasites are typically distinguished by a reduction in their plastomes [18,36,38]. In our study, we observed a significant variation in plastome size and structure among parasitic plants in the Orobanchaceae family. Interestingly, hemiparasitic species of this family exhibited identical results to those of non-parasitic plants. Contrarily, most of the species from other parasitic families displayed a reduction in their plastomes, with the most noteworthy being Convolvulaceae for its highly reduced plastome size. Similar findings were also reported in prior research, which showed that plastomes of most hemiparasitic species within the Orobanchaceae family resembled those of autotrophic plants in terms of genome size, GC content, and gene contents [39]. The stable plastome structure observed in these hemiparasitic plants suggested that their plastomes were in the early stages of transitioning from autotrophic to holoparasitic forms [18]. In our study, it was noteworthy that the Orobanchaceae genomes retained all the *ndh* genes, unlike the genomes of other families where these genes were lost. Additionally, only two species, *V. crassulae* and *V. album* from the Santalaceae family, retained all *ndh* genes, while *S. album* and *S. americana* retained only a few of these genes. Similarly, in a previous study, it was found that the facultative hemiparasite *T. versicolor* (Orobanchaceae) did not lose any plastid genes, though the obligate hemiparasites *S. americana* and *S. hermonthica* from this family exhibited minimal pseudogenization and gene loss, affecting only a few *ndh* genes responsible for encoding subunits of the plastid NAD(P)H dehydrogenase complex. Additionally, in line with our findings, previous research has shown that Santalales hemiparasitic plants are generally characterized by a reduction in their plastome sizes [18,36,38] and previously reported Santalaceae species *D. varians*, *H. parasitica*, and *Macrosolen cochinchinensis* have experienced functional losses of all *ndh* genes. Similarly, in hemiparasitic mistletoes (Viscaceae), their plastomes have undergone slight degradation, resulting in both a reduction in size (down to 126–147 kb) and the loss of all 11 *ndh* genes, which is an identical phenomenon that has been reported in the hemiparasitic *Cuscuta* species [40]. These observations strongly support the concept that functional loss of *ndh* genes represents the initial stage in the transition from autotrophic to parasitic lifestyles [10,15,41]. Plastid ndh genes encode components of the thylakoid NAD(P)H dehydrogenase complex, crucial for stress tolerance but largely dispensable under favorable conditions, with limited significance in modern plants [42,43]. Their absence is common in photoautotrophic seed plants, particularly in parasitic and mycoheterotrophic groups that rely less on photosynthesis, representing an early stage of plastome degradation in heterotrophic plants [37,44,45,46,47]. Independent losses of the ndh complex suggest that carnivorous plants may mitigate environmental stress or reduce reliance on the ndh complex due to prey-derived nutrients, reflecting the evolutionary pathways of carnivory in angiosperms [39,48,49].

In contrast, the plastomes of non-photosynthetic holoparasites typically undergo extensive degradation compared to those of hemiparasites. This degradation not only affects the complete set of photosynthetic genes but also impacts many genes that are not directly related to photosynthesis [10,37,50,51]. Collectively, the comprehensive evidence gathered from various parasitic plastomes strongly indicates a correlation between the extent of plastomic degeneration and the level of heterotrophic dependence among these plants. Generally, genome reduction and multiple rearrangements were predominantly observed in holoparasitic species broadly discussed in previous research [10,18]. In the current study, 113 parasitic plant species, the holoparasitic species of the Convolvulaceae, Apodanthaceae, and Orobanchaceae families, underwent significant genome downsizing. This finding aligns with previous research on various parasitic plant families, highlighting the consistent pattern of genome reduction in holoparasitic species. Our finding is well-supported by earlier work on holoparasitic species (Orobanchaceae) containing plastomes showcasing significant degradation [10,37,44,51,52], with *C. americana* standing out as an extreme example, possessing a plastome of only 46 kb and retaining only 21 intact PCG. Holoparasitic species in Orobanchaceae display a substantial functional or physical loss of genes, including those involved in photosynthesis (*atp*, *ndh*, *psa/b*, *ycf3/4*, *rbc*L, *pet*, *ccs*A, and *cem*A) and housekeeping functions (*rpo* and *rpl/*s), as well as tRNA genes. Similar levels of degradation have been observed in the plastomes of other holoparasitic plants [10,37,44,51,52]. Indeed, *Pilostyles* (Apodanthaceae) exhibits an even more dramatic example of genomic reduction, with its plastomes reduced to a mere 11–15 kb in size with potentially five or six functional genes [11]. Additionally, in certain holoparasitic species like *Rafflesia lagascae* (Rafflesiaceae), the entire plastome may have been completely lost [53].

In the transition from autotrophic to parasitic lifestyles, aside from the degradation of the *ndh* genes, it is also common to observe the loss or pseudogenization of *inf*A in the plastomes of many holoparasitic plants [10,18,37]. As previously reported, *inf*A has been identified as one of the most mobile plastid genes in flowering plants, with a tendency to be transferred to the nucleus [54,55]. In our study, the *inf*A gene was lost in Convolvulaceae, Apodanthaceae, and some species of Santalaceae and Loranthaceae. Other genes like *acc*D and *clp*P were retained by all studied species except Apodanthaceae, while the *ycf1* and *ycf2* genes were lost in Santalaceae, Loranthaceae, and Apodanthaceae, but retained in the remaining cp genome. Similarly, it is important to note that *acc*D plays a crucial role in fatty acid biosynthesis, while *clp*P is not only a potential protease but also participates in the import of proteins into the plastid [56]. Additionally, *ycf1* is essential for plant viability due to its involvement in photosynthetic protein import, being a vital component for the survival of green plants [57], while the function of *ycf2* in plastids remains unknown. These genes have been regarded as essential components of plastomes in non-photosynthetic plants [56] despite their loss in certain cases [39,52]. According to previous literature, *mat*K is associated with RNA processing in plastids [58], while the *rpo* gene, including *rpo*A, *rpo*B, *rpo*C1, and *rpo*C2, plays a crucial role in chloroplast development and function [59]. The absence of the *rpo* gene can have a significant impact on protein synthesis within the chloroplast, consequently influencing the development of chloroplasts and photosynthesis [60]. In this study, the *matK* gene was only lost in holoparasites of Apodanthaceae and Convolvulaceae, while the *psal* and *rpo* (*rpo*A, *rpo*B, *rpo*C1, and *rpo*C2) genes were lost in holoparasites and some hemiparasites of Apodanthaceae, some Orobanchaceae and Convolvulaceae, Cytinaceae (*C. hypocistis*), and Aristolochiaceae (*H. visseri*). The loss of these genes can significantly reduce the capacity of chloroplasts to perform photosynthesis and produce essential materials, thereby making the plant more parasitic in nature. Additionally, the ribosomal protein genes were retained in almost all studied cp genomes. Moreover, plastid genes encoding components of the photosystem (*psa/psb*), the cytochrome complex (*pet*), and heme attachment (*ccs*) are crucial for photosynthesis [14,18]. In holoparasitic angiosperms, the loss or pseudogenization of core photosynthesis genes has been frequently observed [18,61,62]. According to the model of reductive plastome evolution associated with parasitism [18,37], it is suggested that functional losses of photosynthesis-associated genes, such as *psa/psb*, *pet*, *ccsA*, and *cem*A, often occur as plants transition to holoparasitism, marking a critical point in their evolutionary adaptation to a fully parasitic lifestyle. In this study, there was a substantial loss of *atp*, *psa*, *psb*, *pet*, and *rbcL* genes in holoparasitic species of Apodanthaceae, Orobanchaceae (*P. purpurea*, *P. ramose*, *D. coccinea*, *C. phelypaea*, *C. americana*, and *E. virginiana*), Boraginaceae (*P. arenarium*), Aristolochiaceae (*H. visseri*), Cytinaceae (*C. hypocistis*), and Convolvulaceae (*C. strobilacea*, *C. boldinghii*, *C. erosa*, *C. Africana*, and *A. fasciculatum*). The extensive loss of photosynthesis-related genes represents a significant milestone in the evolution of parasitic plastomes [15,63]. Due to their crucial role in photosynthesis, it is imperative for further studies to investigate whether these genes have been functionally transferred to the nuclear genome. This information would provide valuable insights into the mechanisms underlying the transition from autotrophy to parasitism in plants [64].

In angiosperm plastomes, the expansion and contraction of inverted repeats (IRs) are common events [65,66]. In the current work, dramatic IR shifts noted in the plastomes of both holoparasites and hemiparasites suggest that the evolution of parasitism has caused prominent structural changes in the plastomes. These results are in line with those of previous work [19,36,46,52,61,62,67,68,69,70,71,72,73,74,75,76,77,78]. Furthermore, divergence among genes within different parasitic families was investigated and it was found that within the Convolvulaceae family, the *acc*D and *ycf1* genes displayed more divergence (Figure 5B). Additionally, the *ccs*A, *psb*M, *rpl22,* and *atp*F genes showed noteworthy divergence in Loranthaceae, while in Orobanchaceae, the *rpl33*, *rps15*, and *rps18* genes revealed significant divergence, as illustrated in Figure 5. Similarly, the *rpl16* gene exhibited the greatest divergence among all genes in Santalaceae (Figure 5A).

Repeat elements in plastomes indeed play a critical role in genomic rearrangements and recombination [48,79]. In this study, the Orobanchaceae species stand out with the highest numbers of repeats (including palindromic, reverse, forward, and tandem repeats) among all the studied families, whereas Loranthaceae and Convolvulaceae exhibit the lowest numbers of these repeats (Figure S1). In the current study, repeat density and the degree of parasitism were positively correlated. A similar trend was observed previously [10] in 10 plastomes of hemi and holoparasitic plants and their closest nonparasitic relative. Plastome SSRs (cpSSRs) have been demonstrated to be valuable molecular markers for distinguishing species at lower taxonomic levels. Due to their high polymorphism and stability, cpSSRs are potentially useful markers for various applications in population genetics [80,81,82,83,84]. In the present study, higher numbers of SSRs were reported in Loranthaceae and Santalaceae species, with mononucleotide repeats (A/T) being the most abundant type (Figure S2 and Figure 6). This aligns with previous research, which showed that poly (A/T) SSRs are typically more common than other SSR types in many plant plastomes [85,86,87,88,89,90,91]. Furthermore, our results demonstrated a trend of increasing SSR density as genome reduction proceeds.

The analysis of phylogenetic relationships among parasitic plastomes across diverse plant families has unveiled interesting patterns. Here, we discuss the key findings and their implications for our understanding of plant evolution and adaptation to parasitic lifestyles. First, the clustering of species from distinct families, such as Loranthaceae and Schoepfiaceae, suggests that certain evolutionary constraints or shared genomic features may underpin their parasitic lifestyles. Despite the vast phylogenetic distance between these families, their convergence into a single clade indicates a degree of functional similarity possibly driven by adaptation to parasitism. Further investigation into shared genetic elements or physiological mechanisms among these species could provide valuable insights into the molecular basis of parasitism. Additionally, the clustering of holoparasitic plants from the Convolvulaceae family into a separate clade raises questions about the genetic basis of parasitic adaptation within closely related taxa. The presence of distinct genomic signatures, such as the absence of the *ndh* gene family, highlights the importance of gene loss events in shaping the evolution of parasitic plants. Understanding the functional implications of these gene losses, particularly in relation to photosynthesis and energy metabolism, could elucidate the adaptive strategies employed by parasitic plants to exploit their hosts. Moreover, the unexpected associations between families, such as the clustering of the Santalaceae species with Boraginaceae members, indicate a complex evolutionary relationship shaped by both historical contingencies and convergent evolution. Additionally, the division of Orobanchaceae species into two distinct clades, predominantly comprising parasitic taxa lacking the *ndh* gene family, highlights the importance of genomic rearrangements in driving evolutionary divergence within a single family. Investigating the functional consequences of these genomic changes, particularly in relation to host specificity and nutrient acquisition, could provide valuable insights into the ecological interactions between parasitic plants and their hosts. Finally, the clustering of Apodanthaceae species with distantly related taxa, such as *H. visseri* and *C. hypocistis*, underscores the importance of considering convergent evolution and functional constraints in reconstructing phylogenetic relationships among parasitic plants. Integrating genomic, morphological, and ecological data will be essential for resolving the complex evolutionary histories of these enigmatic organisms and shedding light on the mechanisms driving their diversification and adaptation to parasitic lifestyles.

## 5. Conclusions

This study provides a comprehensive analysis of plastome organization, gene composition, and variation across 113 parasitic plant species from various families. Significant variations in plastome size, GC content, and gene retention were observed, with the Orobanchaceae family exhibiting the largest plastomes and Apodanthaceae the smallest. Gene loss, particularly in the ndh gene family and photosynthetic genes, was widespread across parasitic species, reflecting their parasitic lifestyle. Additionally, gene duplications were noted in several species. Comparative analysis revealed substantial gene divergence within families, highlighting the evolutionary adaptation of parasitic plants. These findings deepen our understanding of plastome evolution in parasitic plants and provide insights into their unique genomic characteristics.

### Limitations of the Study

This study offers important insights into the plastome variation in parasitic plants, but there are some areas that could be explored further in future research. One limitation is that the study focused on a relatively small number of parasitic plant families. Including a wider range of species would help create a more complete picture of plastome evolution. Another aspect that could be improved is the functional understanding of the gene losses and duplications observed in the study future research could investigate the specific roles these genetic changes play in the survival and adaptation of parasitic plants. Since this study used existing plastome sequences, it might not capture all the genetic diversity present in parasitic plants, so incorporating more comprehensive sequencing data would be beneficial. Lastly, combining plastome data with transcriptomic and proteomic information could provide a clearer picture of how these genetic variations impact the biology and function of parasitic plants.

## Data Availability

All the data analyzed in this study are included in the published article and are accessible in the NCBI database, as listed in Table S1.

## Supplementary Materials

The following supporting information can be downloaded at https://www.mdpi.com/article/10.3390/genes15121577/s1, Figure S1. The number of forward, reverse, palindromic, and tandem repeats across parasitic plant plastomes. Figure S2. The number of simple sequence repeats (SSR) across parasitic plant plastomes. Table S1. Characteristics of parasitic plastomes.

## References

[1] Nickrent D. (1997). Onwards. The Parasitic Plant Connection. Southern Illinois University. http://www.parasiticplants.siu.edu.

[2] Heide-Jørgensen H. (2008). Parasitic flowering plants. Parasitic Flowering Plants.

[3] Nickrent D.L. (2002). Parasitic plants of the world. Parasit. Plants Iber. Penins. Balear. Isl..

[4] Kuijt J. (1969). The Biology of Parasitic Flowering Plants.

[5] Colwell A.E.L. (1994). Genome Evolution in a Non-Photosynthetic Plant, Conopholis Americana.

[6] Barkman T.J., McNeal J.R., Lim S.-H., Coat G., Croom H.B., Young N.D., Depamphilis C.W. (2007). Mitochondrial DNA suggests at least 11 origins of parasitism in angiosperms and reveals genomic chimerism in parasitic plants. BMC Evol. Biol..

[7] Westwood J.H., Roney J.K., Khatibi P.A., Stromberg V.K. (2009). RNA translocation between parasitic plants and their hosts. Pest Manag. Sci..

[8] Wu C.-S., Lai Y.-T., Lin C.-P., Wang Y.-N., Chaw S.-M. (2009). Evolution of reduced and compact chloroplast genomes (cpDNAs) in gnetophytes: Selection toward a lower-cost strategy. Mol. Phylogenetics Evol..

[9] Ruhlman T.A., Jansen R.K., Maliga P. (2014). The Plastid Genomes of Flowering Plants. Chloroplast Biotechnology: Methods and Protocols.

[10] Wicke S., Müller K.F., de Pamphilis C.W., Quandt D., Wickett N.J., Zhang Y., Renner S.S., Schneeweiss G.M. (2013). Mechanisms of Functional and Physical Genome Reduction in Photosynthetic and Nonphotosynthetic Parasitic Plants of the Broomrape Family. Plant Cell.

[11] Bellot S., Renner S.S. (2016). The Plastomes of Two Species in the Endoparasite Genus Pilostyles (Apodanthaceae) Each Retain Just Five or Six Possibly Functional Genes. Genome Biol. Evol..

[12] Frailey D.C., Chaluvadi S.R., Vaughn J.N., Coatney C.G., Bennetzen J.L. (2018). Gene loss and genome rearrangement in the plastids of five Hemiparasites in the family Orobanchaceae. BMC Plant Biol..

[13] Stefanović S., Olmstead R.G. (2005). Down the Slippery Slope: Plastid Genome Evolution in Convolvulaceae. J. Mol. Evol..

[14] Wicke S., Schneeweiss G.M., de Pamphilis C.W., Müller K.F., Quandt D. (2011). The evolution of the plastid chromosome in land plants: Gene content, gene order, gene function. Plant Mol. Biol..

[15] Barrett C.F., Davis J.I. (2012). The plastid genome of the mycoheterotrophic Corallorhiza striata (Orchidaceae) is in the relatively early stages of degradation. Am. J. Bot..

[16] Graham S.W., Lam V.K.Y., Merckx V.S.F.T. (2017). Plastomes on the edge: The evolutionary breakdown of mycoheterotroph plastid genomes. New Phytol..

[17] Barrett C.F., Specht C.D., Leebens-Mack J., Stevenson D.W., Zomlefer W.B., Davis J.I. (2014). Resolving ancient radiations: Can complete plastid gene sets elucidate deep relationships among the tropical gingers (Zingiberales)?. Ann. Bot..

[18] Wicke S., Naumann J., Chaw S.-M., Jansen R.K. (2018). Chapter Eleven—Molecular Evolution of Plastid Genomes in Parasitic Flowering Plants. Advances in Botanical Research.

[19] Edlund M., Anderson B.M., Su H.-J., Robison T., Caraballo-Ortiz M.A., Der J.P., Nickrent D.L., Petersen G. (2024). Plastome evolution in Santalales involves relaxed selection prior to loss of ndh genes and major boundary shifts of the inverted repeat. Ann. Bot..

[20] Kohzuma K., Dal Bosco C., Kanazawa A., Dhingra A., Nitschke W., Meurer J., Kramer D.M. (2012). Thioredoxin-insensitive plastid ATP synthase that performs moonlighting functions. Proc. Natl. Acad. Sci. USA.

[21] Fu C.-N., Wicke S., Zhu A.-D., Li D.-Z., Gao L.-M. (2023). Distinctive plastome evolution in carnivorous angiosperms. BMC Plant Biol..

[22] Shi L., Chen H., Jiang M., Wang L., Wu X., Huang L., Liu C. (2019). CPGAVAS2, an integrated plastome sequence annotator and analyzer. Nucleic Acids Res..

[23] Schattner P., Brooks A.N., Lowe T.M. (2005). The tRNAscan-SE, snoscan and snoGPS web servers for the detection of tRNAs and snoRNAs. Nucleic Acids Res..

[24] Kearse M., Moir R., Wilson A., Stones-Havas S., Cheung M., Sturrock S., Buxton S., Cooper A., Markowitz S., Duran C. (2012). Geneious Basic: An integrated and extendable desktop software platform for the organization and analysis of sequence data. Bioinformatics.

[25] Waskom M.L. (2021). Seaborn: Statistical data visualization. J. Open Source Softw..

[26] Kurtz S., Choudhuri J.V., Ohlebusch E., Schleiermacher C., Stoye J., Giegerich R. (2001). REPuter: The manifold applications of repeat analysis on a genomic scale. Nucleic Acids Res..

[27] Benson G. (1999). Tandem repeats finder: A program to analyze DNA sequences. Nucleic Acids Res..

[28] Beier S., Thiel T., Münch T., Scholz U., Mascher M. (2017). MISA-web: A web server for microsatellite prediction. Bioinformatics.

[29] Katoh K., Toh H. (2010). Parallelization of the MAFFT multiple sequence alignment program. Bioinformatics.

[30] Katoh K., Standley D.M. (2013). MAFFT multiple sequence alignment software version 7: Improvements in performance and usability. Mol. Biol. Evol..

[31] Capella-Gutiérrez S., Silla-Martínez J.M., Gabaldón T. (2009). trimAl: A tool for automated alignment trimming in large-scale phylogenetic analyses. Bioinformatics.

[32] Darriba D., Taboada G.L., Doallo R., Posada D. (2012). jModelTest 2: More models, new heuristics and parallel computing. Nat. Methods.

[33] Wilgenbusch J.C., Swofford D. (2003). Inferring evolutionary trees with PAUP. Curr. Protoc. Bioinform..

[34] Rambaut A. FigTree v1.3.1. http://tree.bio.ed.ac.uk/software/figtree/.

[35] Clarke C.R., Timko M.P., Yoder J.I., Axtell M.J., Westwood J.H. (2019). Molecular Dialog Between Parasitic Plants and Their Hosts. Annu. Rev. Phytopathol..

[36] Shin H.W., Lee N.S. (2018). Understanding plastome evolution in Hemiparasitic Santalales: Complete chloroplast genomes of three species, Dendrotrophe varians, Helixanthera parasitica, and Macrosolen cochinchinensis. PLoS ONE.

[37] Wicke S., Müller K.F., DePamphilis C.W., Quandt D., Bellot S., Schneeweiss G.M. (2016). Mechanistic model of evolutionary rate variation en route to a nonphotosynthetic lifestyle in plants. Proc. Natl. Acad. Sci. USA.

[38] Li Y., Zhou J.-G., Chen X.-L., Cui Y.-X., Xu Z.-C., Li Y.-H., Song J.-Y., Duan B.-Z., Yao H. (2017). Gene losses and partial deletion of small single-copy regions of the chloroplast genomes of two hemiparasitic Taxillus species. Sci. Rep..

[39] Tang L., Wang T., Hou L., Zhang G., Deng M., Guo X., Ji Y. (2024). Comparative and phylogenetic analyses of Loranthaceae plastomes provide insights into the evolutionary trajectories of plastome degradation in hemiparasitic plants. BMC Plant Biol..

[40] McNeal J.R., Arumugunathan K., Kuehl J.V., Boore J.L., de Pamphilis C.W. (2007). Systematics and plastid genome evolution of the cryptically photosynthetic parasitic plant genus Cuscuta(Convolvulaceae). BMC Biol..

[41] Fan W., Zhu A., Kozaczek M., Shah N., Pabón-Mora N., González F., Mower J.P. (2016). Limited mitogenomic degradation in response to a parasitic lifestyle in Orobanchaceae. Sci. Rep..

[42] Martín M., Sabater B. (2010). Plastid ndh genes in plant evolution. Plant Physiol. Biochem..

[43] Endo T., Shikanai T., Takabayashi A., Asada K., Sato F. (1999). The role of chloroplastic NAD (P) H dehydrogenase in photoprotection. FEBS Lett..

[44] Cusimano N., Wicke S. (2016). Massive intracellular gene transfer during plastid genome reduction in nongreen Orobanchaceae. New Phytol..

[45] Sun Y., Moore M.J., Lin N., Adelalu K.F., Meng A., Jian S., Yang L., Li J., Wang H. (2017). Complete plastome sequencing of both living species of Circaeasteraceae (Ranunculales) reveals unusual rearrangements and the loss of the ndh gene family. BMC Genom..

[46] Bungard R.A. (2004). Photosynthetic evolution in parasitic plants: Insight from the chloroplast genome. BioEssays.

[47] Wu C.-S., Wang T.-J., Wu C.-W., Wang Y.-N., Chaw S.-M. (2017). Plastome evolution in the sole hemiparasitic genus laurel dodder (Cassytha) and insights into the plastid phylogenomics of Lauraceae. Genome Biol. Evol..

[48] Weng M.-L., Blazier J.C., Govindu M., Jansen R.K. (2014). Reconstruction of the ancestral plastid genome in Geraniaceae reveals a correlation between genome rearrangements, repeats, and nucleotide substitution rates. Mol. Biol. Evol..

[49] Braukmann T.W., Broe M.B., Stefanović S., Freudenstein J.V. (2017). On the brink: The highly reduced plastomes of nonphotosynthetic Ericaceae. New Phytol..

[50] Braukmann T., Kuzmina M., Stefanović S. (2013). Plastid genome evolution across the genus Cuscuta (Convolvulaceae): Two clades within subgenus Grammica exhibit extensive gene loss. J. Exp. Bot..

[51] Wolfe K.H., Morden C.W., Palmer J.D. (1992). Function and evolution of a minimal plastid genome from a nonphotosynthetic parasitic plant. Proc. Natl. Acad. Sci. USA.

[52] Li X., Zhang T.-C., Qiao Q., Ren Z., Zhao J., Yonezawa T., Hasegawa M., Crabbe M.J.C., Li J., Zhong Y. (2013). Complete Chloroplast Genome Sequence of Holoparasite Cistanche deserticola (Orobanchaceae) Reveals Gene Loss and Horizontal Gene Transfer from Its Host Haloxylon ammodendron (Chenopodiaceae). PLoS ONE.

[53] Molina J., Hazzouri K.M., Nickrent D., Geisler M., Meyer R.S., Pentony M.M., Flowers J.M., Pelser P., Barcelona J., Inovejas S.A. (2014). Possible Loss of the Chloroplast Genome in the Parasitic Flowering Plant Rafflesia lagascae (Rafflesiaceae). Mol. Biol. Evol..

[54] Millen R.S., Olmstead R.G., Adams K.L., Palmer J.D., Lao N.T., Heggie L., Kavanagh T.A., Hibberd J.M., Gray J.C., Morden C.W. (2001). Many Parallel Losses of infA from Chloroplast DNA during Angiosperm Evolution with Multiple Independent Transfers to the Nucleus. Plant Cell.

[55] Ahmed I., Biggs P.J., Matthews P.J., Collins L.J., Hendy M.D., Lockhart P.J. (2012). Mutational Dynamics of Aroid Chloroplast Genomes. Genome Biol. Evol..

[56] Krause K. (2011). Plastid genomes of parasitic plants: A trail of reductions and losses. Organelle Genetics: Evolution of Organelle Genomes and Gene Expression.

[57] Kikuchi S., Bédard J., Hirano M., Hirabayashi Y., Oishi M., Imai M., Takase M., Ide T., Nakai M. (2013). Uncovering the protein translocon at the chloroplast inner envelope membrane. Science.

[58] Bazrkar-Khatibani L., Fakheri B.-A., Hosseini-Chaleshtori M., Mahender A., Mahdinejad N., Ali J. (2019). Genetic mapping and validation of quantitative trait loci (QTL) for the grain appearance and quality traits in rice (*Oryza sativa* L.) by using recombinant inbred line (RIL) population. Int. J. Genom..

[59] Ohyama K., Fukuzawa H., Kohchi T., Shirai H., Sano T., Sano S., Umesono K., Shiki Y., Takeuchi M., Chang Z. (1986). Chloroplast gene organization deduced from complete sequence of liverwort Marchantia polymorpha chloroplast DNA. Nature.

[60] Pfannschmidt T., Blanvillain R., Merendino L., Courtois F., Chevalier F., Liebers M., Grübler B., Hommel E., Lerbs-Mache S. (2015). Plastid RNA polymerases: Orchestration of enzymes with different evolutionary origins controls chloroplast biogenesis during the plant life cycle. J. Exp. Bot..

[61] Funk H.T., Berg S., Krupinska K., Maier U.G., Krause K. (2007). Complete DNA sequences of the plastid genomes of two parasitic flowering plant species, Cuscuta reflexa and Cuscuta gronovii. BMC Plant Biol..

[62] McNeal J.R., Kuehl J.V., Boore J.L., De Pamphilis C.W. (2007). Complete plastid genome sequences suggest strong selection for retention of photosynthetic genes in the parasitic plant genus Cuscuta. BMC Plant Biol..

[63] Graham M., Hjorth I., Lehdonvirta V. (2017). Digital labour and development: Impacts of global digital labour platforms and the gig economy on worker livelihoods. Transf. Eur. Rev. Labour Res..

[64] Chen X., Fang D., Wu C., Liu B., Liu Y., Sahu S.K., Song B., Yang S., Yang T., Wei J. (2020). Comparative Plastome Analysis of Root- and Stem-Feeding Parasites of Santalales Untangle the Footprints of Feeding Mode and Lifestyle Transitions. Genome Biol. Evol..

[65] Plunkett G.M., Downie S.R. (2000). Expansion and contraction of the chloroplast inverted repeat in Apiaceae subfamily Apioideae. Syst. Bot..

[66] Chumley T.W., Palmer J.D., Mower J.P., Fourcade H.M., Calie P.J., Boore J.L., Jansen R.K. (2006). The complete chloroplast genome sequence of *Pelargonium × hortorum*: Organization and evolution of the largest and most highly rearranged chloroplast genome of land plants. Mol. Biol. Evol..

[67] Krause K. (2008). From chloroplasts to “cryptic” plastids: Evolution of plastid genomes in parasitic plants. Curr. Genet..

[68] Naumann J., Der J.P., Wafula E.K., Jones S.S., Wagner S.T., Honaas L.A., Ralph P.E., Bolin J.F., Maass E., Neinhuis C. (2016). Detecting and characterizing the highly divergent plastid genome of the nonphotosynthetic parasitic plant Hydnora visseri (Hydnoraceae). Genome Biol. Evol..

[69] Samigullin T.H., Logacheva M.D., Penin A.A., Vallejo-Roman C.M. (2016). Complete plastid genome of the recent holoparasite Lathraea squamaria reveals earliest stages of plastome reduction in Orobanchaceae. PLoS ONE.

[70] Stamatakis A. (2006). RAxML-VI-HPC: Maximum likelihood-based phylogenetic analyses with thousands of taxa and mixed models. Bioinformatics.

[71] Schneider A.C., Chun H., Stefanović S., Baldwin B.G. (2018). Punctuated plastome reduction and host–parasite horizontal gene transfer in the holoparasitic plant genus Aphyllon. Proc. R. Soc. B Biol. Sci..

[72] Wu L., Fan P., Zhou J., Li Y., Xu Z., Lin Y., Wang Y., Song J., Yao H. (2023). Gene losses and homology of the chloroplast genomes of Taxillus and Phacellaria species. Genes.

[73] Su H.-J., Hu J.-M. (2016). The complete chloroplast genome of hemiparasitic flowering plant Schoepfia jasminodora. Mitochondrial DNA Part B.

[74] Cho W.-B., Choi B.-H., Kim J.-H., Lee D.-H., Lee J.-H. (2018). Complete plastome sequencing reveals an extremely diminished SSC region in hemiparasitic Pedicularis ishidoyana (Orobanchaceae). Annales Botanici Fennici.

[75] Chen X., Fang D., Xu Y., Duan K., Yoshida S., Yang S., Sahu S.K., Fu H., Guang X., Liu M. (2023). Balanophora genomes display massively convergent evolution with other extreme holoparasites and provide novel insights into parasite–host interactions. Nat. Plants.

[76] Liu S.-S., Hu Y.-H., Maghuly F., Porth I.M., Mao J.-F. (2019). The complete chloroplast genome sequence annotation for Malania oleifera, a critically endangered and important bioresource tree. Conserv. Genet. Resour..

[77] Park I., Yang S., Kim W.J., Noh P., Lee H.O., Moon B.C. (2018). The complete chloroplast genome of Cuscuta pentagona Engelm. Mitochondrial DNA Part B.

[78] Jiang D., Ma R., Li J., Mao Q., Miao N., Mao K. (2019). Characterization of the complete chloroplast genome of Scurrula parasitica. Mitochondrial DNA Part B.

[79] Asano T., Tsudzuki T., Takahashi S., Shimada H., Kadowaki K.-i. (2004). Complete nucleotide sequence of the sugarcane (*Saccharum officinarum*) chloroplast genome: A comparative analysis of four monocot chloroplast genomes. DNA Res..

[80] Provan J., Powell W., Hollingsworth P.M. (2001). Chloroplast microsatellites: New tools for studies in plant ecology and evolution. Trends Ecol. Evol..

[81] Yang A.H., Zhang J.J., Yao X.H., Huang H.W. (2011). Chloroplast microsatellite markers in Liriodendron tulipifera (Magnoliaceae) and cross-species amplification in L. chinense. Am. J. Bot..

[82] Xue J., Wang S., Zhou S.L. (2012). Polymorphic chloroplast microsatellite loci in Nelumbo (Nelumbonaceae). Am. J. Bot..

[83] Hu Y., Woeste K.E., Zhao P. (2017). Completion of the chloroplast genomes of five Chinese Juglans and their contribution to chloroplast phylogeny. Front. Plant Sci..

[84] Ruhsam M., Clark A., Finger A., Wulff A.S., Mill R.R., Thomas P.I., Gardner M.F., Gaudeul M., Ennos R.A., Hollingsworth P.M. (2016). Hidden in plain view: Cryptic diversity in the emblematic Araucaria of New Caledonia. Am. J. Bot..

[85] Yang Y., Zhou T., Duan D., Yang J., Feng L., Zhao G. (2016). Comparative analysis of the complete chloroplast genomes of five Quercus species. Front. Plant Sci..

[86] Asaf S., Waqas M., Khan A.L., Khan M.A., Kang S.-M., Imran Q.M., Shahzad R., Bilal S., Yun B.-W., Lee I.-J. (2017). The complete chloroplast genome of wild rice (*Oryza minuta*) and its comparison to related species. Front. Plant Sci..

[87] Dong W., Xu C., Li W., Xie X., Lu Y., Liu Y., Jin X., Suo Z. (2017). Phylogenetic resolution in Juglans based on complete chloroplast genomes and nuclear DNA sequences. Front. Plant Sci..

[88] Li X., Li Y., Zang M., Li M., Fang Y. (2018). Complete chloroplast genome sequence and phylogenetic analysis of Quercus acutissima. Int. J. Mol. Sci..

[89] Wang X., Zhou T., Bai G., Zhao Y. (2018). Complete chloroplast genome sequence of Fagopyrum dibotrys: Genome features, comparative analysis and phylogenetic relationships. Sci. Rep..

[90] Ye W.-Q., Yap Z.-Y., Li P., Comes H.P., Qiu Y.-X. (2018). Plastome organization, genome-based phylogeny and evolution of plastid genes in Podophylloideae (Berberidaceae). Mol. Phylogenetics Evol..

[91] Zhou T., Wang J., Jia Y., Li W., Xu F., Wang X. (2018). Comparative chloroplast genome analyses of species in Gentiana section Cruciata (Gentianaceae) and the development of authentication markers. Int. J. Mol. Sci..

