# Supplementary figures and images for: Revealing the Dynamic History of Parasitic Plant Plastomes via Structural Characterization, Comparative Analysis, and Phylogenomics
Source: Genes (Basel). 2024 Dec 8;15(12):1577. doi: 10.3390/genes15121577 (PMC11675660; doi:10.3390/genes15121577)

Repeats in cp genomes

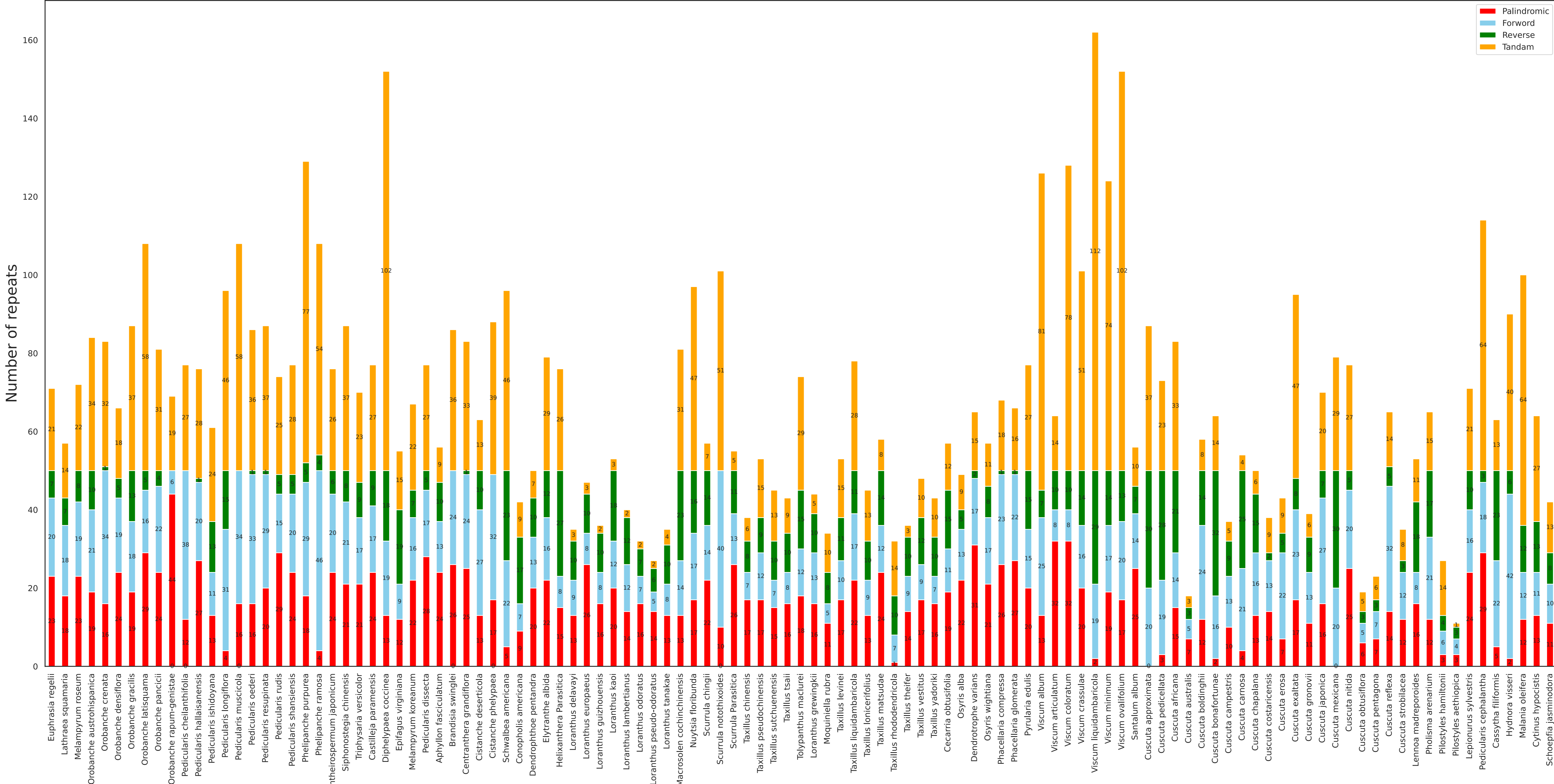

Supplement: Supplementary file 1 [file genes-15-01577-s001.zip › Figure S1.pdf]

SSR in cp genomes

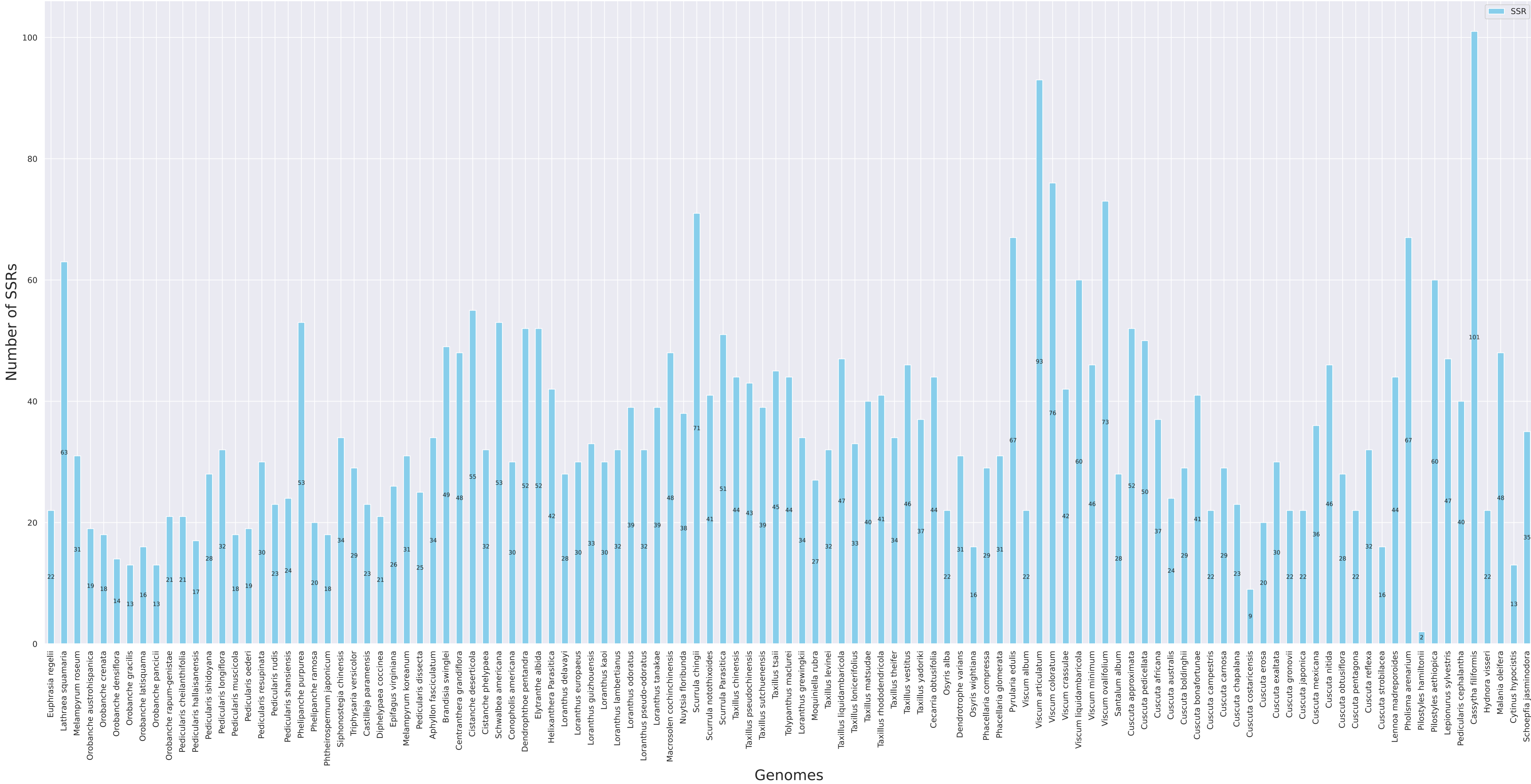

Supplement: Supplementary file 1 [file genes-15-01577-s001.zip › Figure S2.pdf]
